# Long-term follow-up of autologous hematopoietic stem cell transplantation for refractory juvenile dermatomyositis: a case-series study

**DOI:** 10.1186/s12969-018-0284-3

**Published:** 2018-11-20

**Authors:** Jia Zhu, Gaixiu Su, Jianming Lai, Boya Dong, Min Kang, Shengnan Li, Zhixuan Zhou, Fengqi Wu

**Affiliations:** Division of Rheumatology and Immunology, The Affiliated Children’s Hospital, Capital Institute of Pediatrics, Beijing, 100020 China

**Keywords:** Follow-up, Autologous hematopoietic stem cell transplantation, Juvenile dermatomyositis

## Abstract

**Objective:**

To follow up the refractory juvenile dermatomyositis (JDM) with autologous hematopoietic stem cell transplantation (AHSCT) in a long time and to investigate whether AHSCT is effective and safe to treat refractory JDM.

**Methods:**

We collected the AHSCT and follow-up data of three patients with refractory JDM who received autologous peripheral blood CD34+ cell transplantation in our hospital between June 2004 and July 2015. Those data include: hight, weight, routine blood and urine tests, ESR, CK, ALT, AST, LDH, renal functional tests, lymphocyte subpopulations, HRCT and muscle MRI. The last follow-up was done in June 2017.

**Results:**

All three patients had complete remission and could stop prednisone after 3–12 months. None of them relapsed at 144, 113 and 23 months follow-up. Twelve months after their AHSCT, all of their monitoring indexes have returned to normal and they have stopped all medications. Until the date of this article, none of them relapsed or need medicine.

**Conclusion:**

Our study suggests that AHSCT is safe and effective in treating refractory JDM, and it can provides long term drug-free survival. However, more cases are needed for further confirmation.

## Introduction

Juvenile dermatomyositis (JDM) is a chronic autoimmune inflammatory disorder of unknown aetiology that mainly affects muscle and skin. The gold standard treatment for JDM is corticosteroids, along with immunomodulatory therapies, which are used to counteract disease activity, prevent mortality, and reduce long-term disability [1]. Although these medications have led to a significant improvement in prognosis, JDM management remains challenging due to the adverse effects associated with conventional therapies and the occurrence of refractory disease.

Autologous hematopoietic stem cell transplantation (AHSCT) as a treatment for autoimmune diseases (AD) was initiated in 1996, and more than 2000 patients with AD have been treated till 2016. The majority of AD being treated with AHSCT are multiple sclerosis (MS), juvenile idiopathic arthritis (JIA), systemic sclerosis (SSc), Crohn’s disease and so on. There was an overall 85% 5-year survival and 43% progression-free survival. Around 30% of AD patients had complete remission [2]. The major advantage of AHSCT in treating AD is to eliminate the autoimmune T cell cells clones and alter the natural course of the disease [3].

Therefore, we believe that AHSCT could be a therapeutic option for refractory JDM. However, there are only a few case reports about JDM/DM patients who have recovered with successful AHSCT [4–6] and no articles about the long-term results. Herein, we report the treatment and follow-up of three patients with refractory JDM who received AHSCT in our department since 2004.

## Methods

We retrospectively collected AHSCT and follow-up data of three patients with refractory JDM who received autologous peripheral blood CD34+ cell transplantation between June 2004 and July 2015, including height, weight (according to the criteria of standardised curve of growth of Chinese children by Hui et al. [7, 8], routine blood and urine tests, erythrocyte sedimentation rate (ESR), creatine kinase (CK), alanine transaminase (ALT), aspartate aminotransferase (AST), lactate dehydrogenase (LDH), renal functional tests, lymphocyte subpopulations, lung high resolution computed tomography (HRCT), muscle magnetic resonance imaging (MRI) and academic record in their class ranking. Those three patients were the only JDM patients who received AHSCT between June 2004 and June 2017 in our hospital. We searched our medical records with keywords “myositis” and “transplantation”. In June 2017, we did the last follow-up for all of them.

We diagnosed those three patients (2 females, 1 male) with severe and refractory JDM based on the clinical and laboratory criteria proposed by Bohan and Peter [9, 10]. In 2012, the AD guidelines by European Group for Blood and Marrow Transplantation recommended that hematopoietic stem cell transplantation (HSCT) should be considered as a second line therapeutic option or beyond for AD patients with severe progression despite standard and/or approved therapy (level II) [11]. Therefore, all of our three patients met this criteria for HSCT treatment. Some of them failed conventional medication; some had progressive or frequent relapsing disease, which indicated poor prognosis; some had important organs involvement, resulting in a life-threatening condition; and some were intolerable to the toxic side effect of therapeutic drugs.

### Mobilisation and collection of autologous CD34+ cells of peripheral blood

CTX (3–4 g/m^2^) was admitted intravenously for 2 days. Recombinant human granulocyte colony-stimulating factor (G-CSF) 5 μg/(kg.d) was used for mobilisation when WBC decreased to the lowest level. On the fourth day after using G-CSF, we collected mononuclear cells of peripheral blood with a CS-300 blood cell separator,CD34+ cells with CliniMACS cell separator, and removed 3 to 4 logarithmic degree CD3+ cells.

### Preparative regimen

For pretransplant conditioning, high dose CTX (50 mg/kg) was given for 4 days from day − 6 to day − 3. Rabbit antithymocyte globulin (ATG, Saida Company, France) 3.5 mg/(kg•d) was given for 3 days, from day − 4 to day − 2. In day − 2, melphalan 100 mg/(kg•d) was given once. After transplantation of frozen-thawed CD34+ cells on day 0, G-CSF was administered from day 2.

### Treatment and follow-up after transplantation

Immunodepressants were stopped after transplantation, but low doses of glucocorticoids was given continuously and tapered gradually. Antibiotics, such as sulfamethoxine, ganciclovir and fluconazole, were used to prevent infection. IVIG was given monthly for 6 months. The patients were evaluated in the outpatient rheumatology department every 1–3 months for half year, then every 6–12 months till June 2017.

## Results

### The data of three cases

Patient 1 was a 4-year-old male. The onset of the disease was 20 months before the transplantation. He presented with symmetrical proximal muscle weakness, dyspnea, dysphagia and dysphonia. On physical examination, his muscle strength was as following: right lower extremity proximal 1–2/5, distal 3/5; left lower extremity proximal 3/5, distal 4/5; upper extremities proximal 3/5, distal 4/5. He had positive Gottron’s sign and heliotrope rash. His laboratory results showed: CK 1200 U/L (0–195 U/L); negative antinuclear antibody (ANA). Electromyogram (EMG) showed myogenic damage. Muscle MRI showed diffuse muscle involvement of proximal legs. HRCT showed mild pulmonary interstitial disease. A left quadriceps biopsy showed extensive muscle atrophy, focal necrosis, small vessel wall degeneration and thickening, fibrous thrombosis, and fatty tissue hyperplasia. Initially we gave him intravenous immunoglobulin (IVIG) (2.0 g/kg per month for 3 months), cyclophosphamide (CTX) (1 g/m^2^ body surface area monthly intravenously for 6 months), and high-dose methylprednisolone (MP) (20 mg/kg per day for 3 days) followed with prednisone 2 mg/kg daily. After 6 months of treatment, his rash, expiratory dyspnea, dysphagia and dysphonia improved, but muscle weakness remained. Therefore, we recommended AHSCT and he received the transplantation in June 2005.

Patient 2 was a 7-year-old girl. The onset of the disease was 31 months before AHSCT. Her symptoms included fever, muscle weakness, dysphonia, dyspnea and dysphagia. On physical examination, her muscle power was as following: right lower extremities proximal 2/5, distal 4/5; upper extremities proximal 3/5, distal 3/5. Gottron’s sign is positive. Her laboratory results showed serum CK 500 U/L (0–195 U/L) and negative ANA. EMG showed myogenic damage. Muscle MRI showed diffuse muscle enhancement of proximal legs and limbs. HRCT showed severe pulmonary parenchyma and interstitial disease. A right quadriceps biopsy showed denatured, broken and dissolved muscle, along with focal chronic inflammatory cells and positive Masson staining. We intubated her and placed her on a ventilator, and simultaneously gave her IVIG (2.0 g/kg per month•3 months), CTX (1 g/m^2^ body surface area intravenously monthly for 6 months), high-dose MP (20 mg/kg daily for 5 days) and followed by prednisone 2 mg/kg daily. Two weeks later, her dyspnea improved, and tracheal intubation was removed. One month later, her dysphagia and dysphonia improved. But the improvement of muscle weakness and rash was not obvious. So we gave her methotrexate (MTX) 15 mg/ m^2^ body surface area and cyclosporine A (CsA). Nine months after the initial treatment, her muscle weakness and rash were not improved. Therefore, we recommended AHCST and she received the transplant in January 2008.

Patient 3 was a two and half years old female. The onset of JDM was 6 months before the transplant. She presented with muscle weakness and dysphagia. On physical examination, her muscle strength was as following: lower extremities proximal 2/5, distal 3/5; upper extremities proximal 2/5, distal 3/5. Gottron’s sign was positive. Her laboratory tests showed high serum CK 2569 U/L (normal: 0–195 U/L) and negative antinuclear antibody. EMG showed myogenic damage. Muscle MRI showed diffuse muscle involvement of proximal legs. HRCT showed spot shadow in left lung, and focal interlobular septal thickening. Parents refused muscle biopsy. Initially, we gave her IVIG (2.0 g/kg per month•3 months), CTX (1 g/m^2^ body surface area intravenously monthly for 5 months), high-dose MP (20 mg/kg daily • 5 days), followed by prednisone 2 mg/kg daily. After 5 months of treatment, her rash and dysphagia improved, but muscle weakness remained the same. Therefore, we recommended AHCST, and the girl received the transplant in July 2015.

### Reconstitution of hematopoietic function

Three to eight days after the AHSCT, the leukocyte and lymphocyte levels of all 3 patients decreased to the lowest level (0.01 × 10^9^/L). The platelets decreased to 5–10 × 10^9^/L. Haemoglobin (HGB) decreased to 30–60 g/L. 10 to 14 days after AHSCT, the neutrophils increased to more than 1.0 × 10^9^/L. 14 to 16 days after the AHSCT, the platelets came back to 20 × 10^9^/L. Those results indicated that AHSCT for all three patients were succeeded.

### Follow-up of patients’ condition

#### Immunological reconstitution

**Fig. 1:**
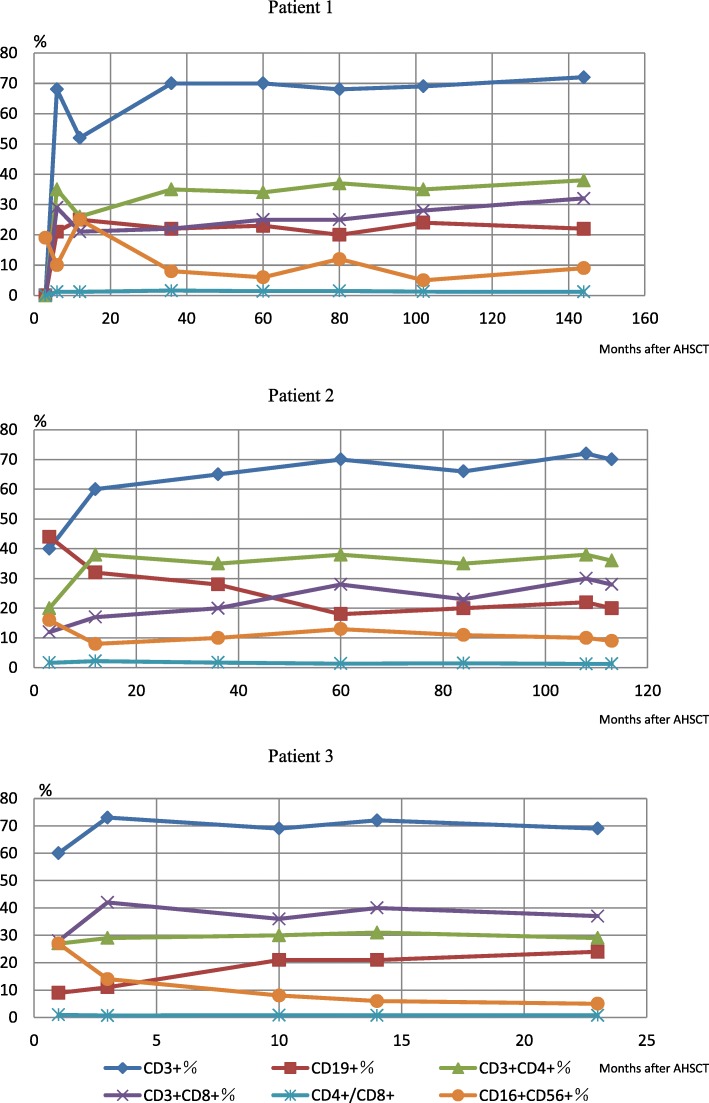
The proportion of different cells in peripheral blood of three patients after AHSCT

The immunological function was obviously inhibited after the auto-PBHSCT (Fig. 1). The number of CD4 and CD8 cells remained low within 3 months after the transplantation. 6 months later, the number of CD4+ and CD8+ cells returned to normal.

#### Clinical manifestation

In the first 6 months after AHSCT, muscle weakness and rash improved slowly for all three patients. However, after 6 months, the improvement was much faster. About 12 months later, all of their monitoring indexes, including immunological function, CK, AST, height, weight and academic record in their class ranking, returned to normal without taking any medication. They remained stable without relapse till this article was written.

**Fig. 2:**
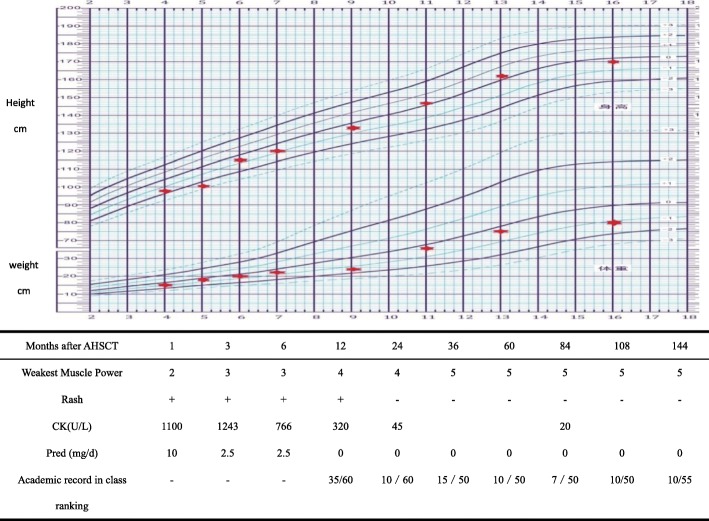
Patient 1’s height, weight and other clinical characteristics after AHSCT

Patient 1 has been followed up for 144 months. He didn’t show any sign of JDM relapsing, and his immunological function was normal. All of his medications were stopped after HSCT. At last follow-up, his height was 170 cm, and body weight was 50 kg. He went to school as an ordinary student and performed well in class (Fig. 2).

**Fig. 3:**
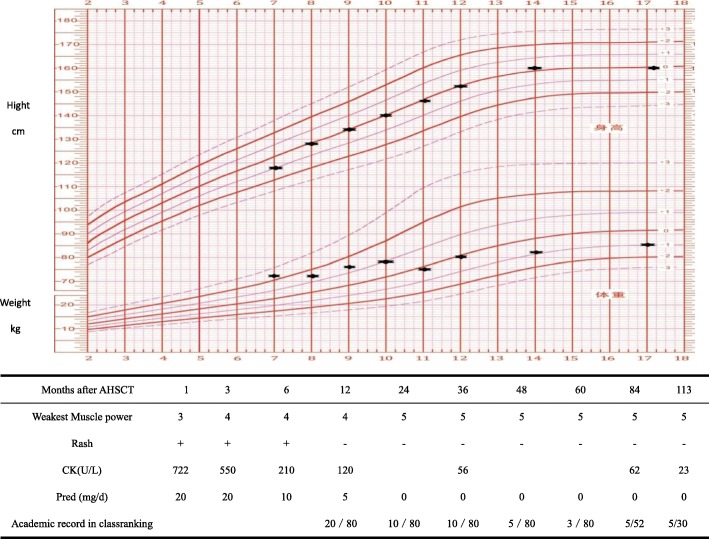
Patient 2’s height, weight and other clinical characteristics after AHSCT

Patient 2 has been followed up for 113 months. During that time, she had no sign of JDM activation. Her immunological function was normal, and she did not take any medication anymore. Her height was 160 cm, and her weight was 42 kg. Her menarch was at 14, and period was regular, 5–7/30 days, with normal volume. She went to school as others and performed well in class (Fig. 3).

**Fig. 4:**
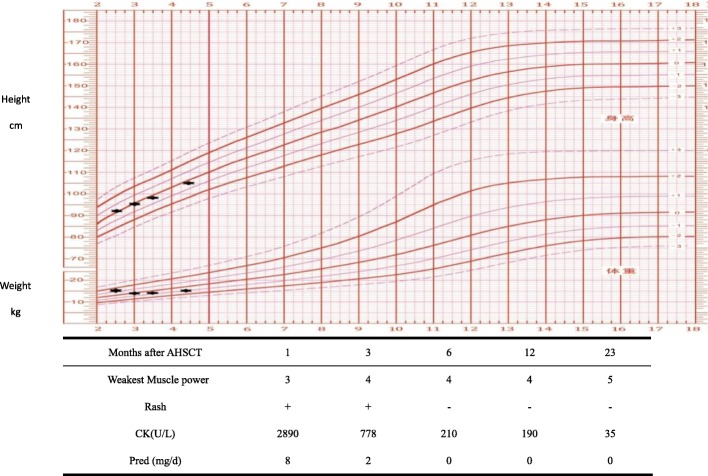
Patient 3’s height, weight and other clinical characteristics after AHSCT

Patient 3 has been followed up for 23 months. She had no sign of JDM activation. Her immunological function was normal, and she did not take any medication anymore. Her height was 105 cm, and her weight was 15 kg. She went to kindergarten, and performed well (Fig. 4).

#### Transplantation-related complications

Patient 1 had fever and cough because of Epstein–Barr virus infection 7 days after the transplantation. He was cured with 3-week introvanous ganciclovir. Patient 2 had no complication after the transplantation. Patient 3 had CMV infection 32 days after the transplantation and recovered with 5-week introvanous ganciclovir. No other complications were found in our three patients. There were no deaths and no graft-versus-host disease in our cases.

## Discussion

Our three patients presented with a typical cutaneous change of JDM with diffuse muscle involvement. Since they were not cured by conventional immunosuppressive, they all met the creation for refractory JDM. Till now, it is very difficult to treat refractory JDM. Many researchers have contributed to find out new treatment. In 2011, Pediatric Rheumatology International Trials Organisation (PRINTO) studied 145 patients with recent-onset JDM and 130 patients with one disease flare. It found out that cyclosporine and IVIG were preferred for treating relapse in Europe [12]. In 2012, the Childhood Arthritis and Rheumatology Research Alliance (CARRA) put forward three consensus protocols, including steroids, methotrexate and IVIG, in order to optimize the baseline standard therapy for patients with moderate to severe JDM [13]. However, those medications have unavoidable toxic side effects, such as weight gain, growth delay, gonadoinhibitory and so on. Therefore, we would like to find out a new way to cure refractory JDM, based on its possible pathogenesis. Although the aetiology of JDM remains unclear, current theories propose a combination of environmental triggers, immune dysfunction and specific tissue responses as possible causes [14]. Many literatures point out that JDM is a true inflammatory small vessel vasculitis, and cytokines, such as interferons and tumor necrosis factor α, play an important role in the pathogenesis [1, 15]. Besides, literatures showed AHSCT was safe for children [7, 16]. Therefore, we thought that autologous HSCT might be effective and safe for refractory JDM.

Autologous HSCT was first reported to treat DM/PM in 2000 [17]. Until now, only 11 cases were reported (Table 1), and 5 of them were children. All of these patients had tried a lot of conventional drugs before HSCT and some of them tried biotherapy (4 cases used rituximab, 2 cases used infliximab, 1 case used alemtuzumab) without any improvement. All of our three patients had tried conventional medicatons for 6 to 31 months before HSCT, but none of them used biologics. During 2005–2008, biologics was rarely used in China, and its efficacy in dermatomyositis is not confirmed [15].Therefore, our three patients did not try biologics. [4–6, 17–21]. After AHSCT, 13 cases had improved (13/14), and only one case (1/14) did not. There was no death. Therefore, we and articles verified together that AHSCT was an effective and safe way to treat refractory DM/PM patients.

**Table 1:** The clinical characteristics of ten cases of DM/PM treated with AHSCT in articles

First Author/Year	diseases	*n*	Gender	age	Treatment before HSCT^a^	Mobilization	Conditioning	Improve	Follow-up (months)	complication
Baron, 2000 [17]	PM	1	Female	28	Pred, AZA, MTX, CTX	CTX + G-CSF	ATG, CTX	yes	12	severe ARDS; fevers and chills by ATG
Chakraverty,2001 [4]	DM	1	Male	46	DEX, vincristine, adriamycin	CTX + G-CSF	melphalan	yes	18	severe mucositis
Bingham, 2001 [21]	PM	1	Female	38	Pred, CTX, CsA, AZA, IVIG, plasmapheresis	CTX + G-CSF	–	yes	24	no
Oryoji, 2005 [5]	DM	1	Female	54	Pred, CsA, CTX	CTX + G-CSF	CTX	Yes	12	Cytomegalovirus antigenemia
Henes, 2009 [18]	PM	1	Male	32	Pred, AZA, MTX, CsA, MMF, IVIG, CTX, alemtuzumab, RTX, IFX	CTX + G-CSF	CTX, ATG	Yes	36	No
Holzer, 2010 [19]	JDM	2	Female	12	Pred, MTX, CsA, IVIG, RTX	CTX + G-CSF	ATG, CTX,fludarabine	yes	26	No
				8	Pred, MTX, CTX, RTX, tacrolimus	CTX + G-CSF	ATG, CTX,fludarabine	Yes	13	No
Storek, 2013 [6]	DM	1	Male	22	Pred, IVIG, AZA, MMF, CTX, CsA, IFX, RTX	CTX + G-CSF	CTX, ATG	no	3	No
Enders,2015 [20]	JDM	3	–	–	–	–	–	Yes	36–60	–

^a^*Pred* is prednisone, *CTX* is cyclophosphamide, *IVIG* is intravenous immunoglobulin, *MTX* is methotrexate, *CsA* is cyclosporine A, *RTX* is rituximab, *IFX* is infliximab, *MMF* is mycophenolate mofetida

Stem cells were mobilized by application of 2 g/m^2^ cyclophosphamide and subsequent administration of granulocyte colonystimulating factor (G-CSF) in our cases, the same as in reported articles. Chakraverty chose DEX, vincristine and Adriamycin, because his patient had combined multiple myeloma [4]. The conditioning regimens were different among articles. However, CTX and ATG were included in almost all regiments. For our three cases, we used CTX, ATG and melphalan as the conditioning regimen, because my first patient (patient 1) received AHSCT in 2005. At that time, no successful case was reported about JDM treated by HSCT. Since our patient responded poorly to immunosuppressants, we gave him an intensive conditioning. All of our three patients were successfully transplanted and improved. After AHSCT, patient 1 and patient 3 developed virus infection, but no other complication or death. According to articles (Table 1), reported complications include three virus infection (3/11), one mycobacterium avium infection (1/11), one acute respiratory distress syndrome (1/11), one ATG allergy (1/11) and one mucositis (1/11). 5 out of 10 patients had no complication after transplantation. No death was reported (0/14). Therefore, we concluded that AHSCT would be a safe treatment for DM/PM.

Our follow-up time was the longest in the literatures. After AHSCT, our 3 patients only received oral prednisone for 3–12 months. Their muscle weakness and rash improved gradually. Twelve months later, all of their monitoring indexes returned to normal and they are not taking any medications. Until this article was written, they all stayed medication free and had no relapse. In 2010, the European Group for Blood and Marrow Transplantation released a long-term follow-up result, including SSc, lupus and JIA, but no exact data of DM/PM. It showed that the 5-year overall survival rate was more than 76%, the transplant related mortality was less than 11% [22]. In 2017, the European Society for Blood and Marrow Transplantation Autoimmune Diseases Working Party recommended that AHSCT should be considered after careful evaluation of patients’ medical condition [23]. However, these articles did not clarify whether those patients had long-term immunosuppressive medications or glucocorticoids after transplantation. Our study could show AHSCT is able to cure refractory DM, and may provide long-term drug free survival.

Our study still had some limitations. Firstly, we did not do validated clinical scores, and we just focused on the weakest muscle, which makes it a little difficult to compare with other studies of treatments in this field. Secondly, the follow-up time of the three children was not identical. We will improve our work in future.

## Conclusion

Treatment of refractory JDM is challenging. Long-term use of corticosteroids and immunosuppressive agents may cause severe adverse reactions and psychological problem in children. Although there were only a few articles about AHSCT in treating JDM, our study supports that this method could be an effective and safe to treat refractory JDM and provide long-term drug free survival. However, we will need more clinical data to confirm it in the future.

## Abbreviation

AD: autoimmune diseases
AHSCT: autologous hematopoietic stem cell transplantation
ALT: alanine transaminase
ANA: antinuclear antibody
AST: aspartate aminotransferase
ATG: antithymocyte globulin
CARRA: Childhood Arthritis and Rheumatology Research Alliance
CK: creatine kinase
CsA: cyclosporine A
CTX: cyclophosphamide
EMG: Electromyogram
ESR: erythrocyte sedimentation rate
G-CSF: granulocyte colony-stimulating factor
HGB: Haemoglobin
HRCT: high resolution computed tomography
HSCT: hematopoietic stem cell transplantation
IVIG: intravenous immunoglobulin
JDM: juvenile dermatomyositis
JIA: juvenile idiopathic arthritis
LDH: lactate dehydrogenase
MP: methylprednisolone
MRI: magnetic resonance imaging
MS: multiple sclerosis
MTX: methotrexate
PRINTO: Pediatric Rheumatology International Trials Organisation
SSc: systemic sclerosis
